# Integration of Different Disciplines in Medicine: A Vertical Integrated Teaching Session for Undergraduate Medical Students

**DOI:** 10.30476/jamp.2020.87082.1289

**Published:** 2020-10

**Authors:** AMIT KUMAR MISHRA, ROSELIN MOHANDAS, MANIKANDAN MANI

**Affiliations:** 1 Department of Community Medicine, Pondicherry Institute of Medical Sciences, Kalapet, Pondicherry 605014, India; All the authors equally contributed in the intellectual content, conception and design of this work, the analysis and interpretation of the data, as well as in the writing & reviewing of the manuscript

**Keywords:** Medical education, Teaching, Medical, Feedback

## Abstract

**Introduction:**

The medical course is very vast and disciplines are covered in different phases. Most of the teaching methods are didactic and conducted by individual disciplines; that’s why students fail to see the relevance of different disciplines and do not develop the required skills such as critical thinking, problem solving, and decision making. Solution to this is integrated teaching by various disciplines.

**Methods:**

A cross-sectional study was conducted among VI semester MBBS students and all the students who attended the class were included, using universal sampling. A descriptive analysis of the feedback was done for the students at the end of an integrated teaching session to assess the perceptions of students towards a new method of integrated teaching. A handout with all the relevant information was shared with the students as the study material. The results were presented in percentages.

**Results:**

Ninety seven percent of the students stated that the objective of the session was achieved. Most of the students (92.7%) believed that they had learned new skills which would be applicable in future practice. Most of the students (42.3%) appreciated the problem solving session followed by all the sessions equally (20.7%). Around 97.3% of the students shared that the handouts produced them with valuable information to support the session, and 92.8% of them gave feedback that they would use it in future as a reference material.

**Conclusion:**

The current descriptive analysis shows that students appreciated and enjoyed this new method of teaching learning session with the problem solving section as the most appreciated part of the integrated teaching session. The handout was well appreciated and utilized as a reference material during the session and students were also interested in using the same in future as a reference material.

## Introduction

The medical course is very vast and disciplines are covered in different phases. Most of the teaching methods are didactic and conducted by individual disciplines. In isolated discipline teaching, students fail to see the relevance of different disciplines ( 1 , 2 ) and do not develop the required skills such as critical thinking, problem solving, and decision making ( 3 ). That is why the students get discouraged from active learning and disinterested in transforming the knowledge into practice ( 4 ). The current need of medical education necessitates both an understanding of interconnectedness between different subjects and their application in providing patient care ( 5 ). Therefore, there is a need to teach the students by correlating various disciplines to create interest and promote active learning ( 6 ), and one of the ways to address this issue is integrated teaching by various disciplines. Integration is defined as organization of teaching matter to correlate different subjects usually taught in separate academic phases ( 7 ) or coordination in the teaching learning activities to ensure harmonious functioning of the educational processes ( 8 ). The Medical Council of India (MCI) has incorporated integration of medical curriculum for teaching undergraduate medical students with the specific objective of providing knowledge in a holistic way ( 5 ). Horizontal integration is integration of disciplines in the same phase, while vertical integration is an integration between different disciplines taught in different phases of medical syllabus. One of the advantages of this integrated curriculum is a good perception of the learning environment ( 9 ) as different subject experts deliver their topics in a correlated fashion. Though the integrated teaching sessions were conducted in UG teaching previously; now as per the new MCI curriculum, it is part of the routine teaching training programme of undergraduate students with more specific learning objectives and micro-planning. Integrated teaching does not only mean integration of disciplines, but also it should be a micro-planned well-conducted series of sessions which includes all the three domains of learning: cognitive, affective, and psychomotor ( 10 ). Faculty can use new methods of teaching learning methods during the integrated teaching sessions to make it more interesting, but at the same time the students’ feedback regarding a new teaching method is of paramount importance as the feedback will help to improve the learning sessions. Keeping all these facts in mind, we performed an analysis of the feedback collected during a vertical integrated session with a new teaching method to understand the perceptions of students on integrated teaching.

## Methods

A vertical integrated teaching session was conducted for the VI semester MBBS students which was coordinated by the Department of Community Medicine. Based on the topic selected for the integrated teaching class, i.e. Nutrition among Tuberculosis (TB) patients and children with acute malnutrition, the following departments were planned to get involved in the integrated teaching session: Pulmonary Medicine, Paediatric, Dietetics Department. The above- mentioned topic was selected as both Tuberculosis and Malnutrition are highly prevalent diseases in India and as a health care provider in future students should know about the diseases in more detail. As per the request from the organizing department, a faculty from each department was deputed for the teaching session. A meeting was conducted with the designated faculty for micro-planning the integrated teaching session and the lesson plan was accordingly made. A handout with all the relevant information required for the session was prepared with reference to National Guidelines of India and World Health Organization (WHO) pertaining to nutrition among Tuberculosis patients and children with acute malnutrition. The handout was shared with the students 2 days before the scheduled integrated teaching session.

**Figure 1:**
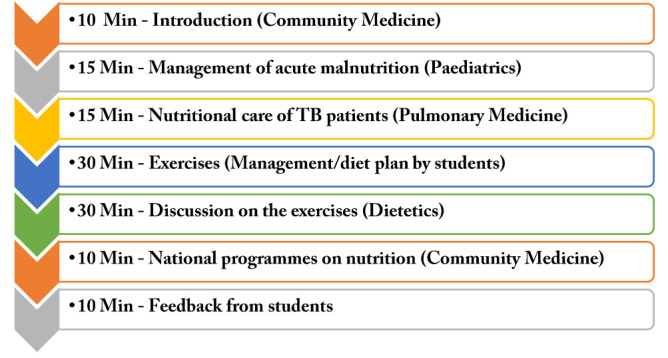
Lesson plan for the integrated teaching session

On the day of the session, all the students present were divided into small groups (6-7 students in each group) and sitting arrangements were made accordingly. The lesson plan was presented to them. The whole session was divided into 3 phases: the first phase was on basics of nutrition in these 2 common diseases, the second one was the problem solving/exercises by the students as per the discussion in the previous phase, and the third phase was discussion on the problems/exercises by the subject experts. The sessions on introduction (epidemiology), management of acute malnutrition and nutritional care of TB patients were taken by the deputed faculties from various departments as per the lesson plan (Figure 1). After the above sessions on basics of nutrition, case scenarios related to the management/diet plan for a case of TB and a child with acute malnutrition were presented on the screen. The students with the basic ideas from the first phase and with the help of the study material (handout) shared to them tried to plan the diet of the case exercises given to them within a specific time period. The students in the respective groups discussed among themselves and planned a simple diet (breakfast to dinner) for the patients in the case scenarios. In the next session, the diet plan for the cases were discussed in detail by a dietician involved in patient care in a tertiary hospital with the help of subject experts, pulmonologist and paediatrician. To complete the topic in discussion, the National Health Programmes related to the case scenarios were discussed briefly. As the current method was planned and implemented the first time during an integrated teaching session, at the end of the integrated teaching session feedback from the students was collected in a structured printed format. Due to the lack of time pre- and post- tests could not be done. The feedback obtained was anonymous and collected by a single person before the students left the class. 

The collected data were entered in EpiData (The EpiData Association, Odense, Denmark) and data analysis was done by SPSS Statistic Package V.21 (developed by IBM Corp, Armonk, New York). The results were presented in percentages and proportions. 

## Results

The integrated teaching session was attended by 111 final year (Part - I MBBS) undergraduate students and 108 (97.3%) students shared that the objectives as told in the lesson plan during the introduction section of the integrated teaching session were achieved at the end of the session.

The students were asked to rank the feedback on the integrated session on a scale of 1 to 5, where 1 was poor and 5 was excellent. Among all the students, 99 (89.2%) of the students commented that the use of audio-visual aids was good and above (≥ 3). Most of the students ranked 3 and above, i.e. good, very good, and excellent for different aspects of the integrated teaching session such as the use of various teaching aids, clarity of communication, proper management of time, overall organization of the teaching session, and the skills they learned. Very few students ranked these aspects as poor and fair in their feedback at the end of the session (Table 1).

**Table 1:** Feedback of students on execution of the session

Feedback	Poor n (%)	Fair n (%)	Good n (%)	Very good n (%)	Excellent n (%)
Use of various teaching aids	1 (0.9)	11 (9.9)	32 (28.8)	36 (32.4)	31 (27.9)
Clarity of communication	-	15 (13.5)	34 (30.6)	42 (37.8)	20 (18)
Proper management of time	6 (5.4)	14 (12.6)	34 (30.6)	33 (29.7)	24 (21.6)
Overall organization	3 (2.7)	6 (5.4)	33 (29.8)	42 (37.8)	27 (24.3)
Skills learned	1 (0.9)	7 (6.3)	34 (30.6)	47 (42.3)	22 (19.8)

Most of the students, (N=47; 42.3%) appreciated the exercise solving session (practical part, diet calculation); the second phase of the teaching session was followed by all the sections equally (N=23; 20.7%). Six students mostly appreciated the cases scenario discussion by the dietician. Twenty nine students did not respond the question on the mostly appreciated section of the teaching learning session (Table 2).

**Table 2:** Sections of the teaching session mostly appreciated by the students

Section	N (%)
All sessions equally	23 (20.7)
Case scenario discussion	6 (5.4)
Chest Medicine	4 (3.6)
Diet Calculation	47 (42.3)
Nutrition	2 (1.8)
No response	29 (26.1)

Only 7 (6.3%) students went through most of the part of the handout (> 80% of the study material) and 35 (31.5%) students went through one fifth of the study material only (Table 3).

**Table 3:** Proportion of the reading material completed by students before the session

Percentage of the handout you able to cover before the session.	N (%)
<20	35 (31.5)
20-40	18 (16.2)
40-60	34 (30.6)
60-80	17 (15.3)
>80	7 (6.3)

Regarding the handout shared with the students, 109 (98.2%) students gave feedback that the handout was concise, confined to the topic covered during the integrated teaching session and specific information were available; also, 108 (97.3%) students stated that the handout provided them with valuable information to support the exercise solving session and 103 (92.8%) students gave feedback that they would use it in future as a reference material (Table 4).

**Table 4:** Feedback of students on the handout shared with them for the session

Feedback	Yes n (%)	No n (%)
Was the handout confined to the topic?	109 (98.2)	2 (1.8)
Handout produced valuable information to support the session?	108 (97.3)	3 (2.7)
Will you be using the handouts as future reference material?	103 (92.8)	8 (7.2)

Few students gave specific suggestions for future improvement in the teaching learning session to make it more interesting and attractive. Nine students asked to make the session of shorter duration, preferably 1 hour. Six students suggested that a more detailed handout should be prepared and a more detailed discussion should be arranged, preferably small group discussions. Four (3.6%) students suggested that we should include more videos in the session. Six (5.4%) students suggested that hard copies of the handout should be distributed among the students, and two of them suggested that the handout should be made more concisely, simpler and should be shared well in advance for reading (Table 5).

**Table 5:** Specific suggestions by the students

Suggestions	N (%)
Suggestions on integrated teaching session	
No suggestions	87 (78.4)
Session of shorter duration	9 (8.1)
Video presentations	4 (3.6)
Clarity in explanations	2 (1.8)
Detail discussion of exercises	3 (2.7)
More informative handouts, more detailed discussion and exercises	6 (5.4)
Suggestions on handout	
No suggestions	99 (89.2)
Distribution of hard copy of handouts	6 (5.4)
Handout should be more concise	2 (1.8)
Handout should be more simple	2 (1.8)
Distribution ofhandouts well in advance	2 (1.8)

## Discussion

To improve the quality of the students and make them more competent, integrated teaching is needed ( 11 ). Few studies have shown that students who are trained with such an integrated curriculum/medical education use their skills and make a more accurate diagnosis than those trained in the conventional way ( 12 ). Integrated teaching improves the cognitive and psychomotor domains of undergraduate students and creates active interest in topics ( 13 ). As the objective of the integrated teaching is to make the students more competent in providing health care services, and nutritional problem is one of the major national health issues in our country, the topic for the vertical integrated teaching selected was “Nutrition among Tuberculosis patients and children with acute malnutrition”. 

Most of the students appreciated the integrated teaching session and shared that the objectives of the integrated teaching session were achieved; this isin the sa,e line with the studies by Yadav et al. at Gujurat ( 14 ). Khan et al. at Malaysia ( 15 ), and Muthukumar et al. at Pondicherry ( 16 ). Most of the students commented that the time management and use of audio-visual aids were done properly and there was clarity in communication by faculty experts. Students also shared that they had learned new skills which would be applicable in future practice. The findings of the current study are similar to those of the studies conducted by Toppo et al. at Jabalpur, ( 17 ) Dandannavar et al. at Karnataka, ( 18 ) Nikam et al. at Mumbai, ( 19 ) Kadam et al. at Maharashtra, ( 20 ) Soudarssanane et al. at Pondicherry, ( 21 ) Kalapana et al. at Bangalore, ( 22 ) Mahajan et al. at Ahmedabad ( 23 ), and Rehman et al. at Pakistan ( 24 ).

Feedback on mostly appreciated section of the integrated teaching session, when all the students who attended the session were asked, was that most of the students appreciated the exercise solving section/practical followed by all the session equally. During the problem solving section, the students were divided into smaller group and tried to plan a sample diet for the case scenarios given to them. There was active participation of each and every students in their respective small groups similar to a study in North India ( 25 ). The handout prepared for the section was well appreciated by the students and they were interested in using the same as reference material in future. Because of the routine classes and task allotted to them, most of the students could not go through the handout completely before the scheduled integrated teaching class. Only a few students went through the study material before the class. Students gave the feedback that it would be better to share the study materials well in advance, so that they could go through it before the scheduled classes and also suggested to share a hardcopy of the study material as it was more convenient for them than the soft copy. In the current study, 103 (92.8%) students shared that the skills they learnt would be applicable in future practice; this is similar to the findings of a study carried out by Neeli et al. at Andhra Pradesh, where ( 6 ) 117 (91%) students agreed that it provided them with the knowledge and skills that would be helpful in clinical practice. In another similar study at Ahmedabad, the students provided feedback that it gave them the knowledge and skills helpful in clinical practice 75 (93.75%) and it also provided concept clarity 71 (88.75%) ( 26 ).

Around one fourth of the students suggested that we should improve the integrated teaching session and 12 students stated that we need to improve the handout/ study material. Few students suggested that we should reduce the integrated teaching session to one hour, but to cover the whole topic by different disciplines within one hour is not feasible, whereas in a study at Ahmedabad three fifths of the students agreed to have more time allotted for each topic &amp; sixteen percent of them disagreed with it ( 6 ). Six (5.4%) students suggested that they should have more detailed and small group discussions in the current study, which was also suggested in other studies conducted in India ( 6 , 22 ). Students felt that the topic of integrated teaching was relevant to the clinical practice and the skills learnt would be used in future practice, which is similar to the results of the studies conducted by Kalapana et al. ( 22 ) and Ambwani et al. ( 27 ).

One of the limitations of the study is that it was a descriptive study where the results of the new method of integrated teaching could not be compared with the control or conventional study method. The other limitation of the study was that all the students of that batch/ semester could not participate in the integrated teaching session.

## Conclusion

The current descriptive analysis of the feedback received from the students shows that the students appreciated and enjoyed the method used in the current teaching learning session with the practical part, problem solving section as the mostly appreciated part of the integrated teaching session. The handout relevant to the topic which was shared with the students was well appreciated and utilized as a reference material during the session and students were interested in using the same material in future as a reference.
